# The effect of foot orthoses and in-shoe wedges during cycling: a systematic review

**DOI:** 10.1186/1757-1146-7-31

**Published:** 2014-05-23

**Authors:** Boon K Yeo, Daniel R Bonanno

**Affiliations:** 1 Department of Podiatry, Faculty of Health Sciences, La Trobe University, Bundoora, Vic 3086, Australia; 2 Lower Extremity and Gait Studies Program, Faculty of Health Sciences, La Trobe University, Vic 3086, Australia

**Keywords:** Foot orthoses, Wedges, Cycling, Kinetics, Kinematics, Oxygen consumption, Power production

## Abstract

**Background:**

The use of foot orthoses and in-shoe wedges in cycling are largely based on theoretical benefits and anecdotal evidence. This review aimed to systematically collect all published research on this topic, critically evaluate the methods and summarise the findings.

**Methods:**

Study inclusion criteria were: all empirical studies that evaluated the effects of foot orthoses or in-shoe wedges on cycling; outcome measures that investigated physiological parameters, kinematics and kinetics of the lower limb, and power; and, published in English. Studies were located by data-base searching (Medline, CINAHL, Embase and SPORTDiscus) and hand-searching in February 2014. Selected studies were assessed for methodological quality using a modified Quality Index. Data were synthesised descriptively. Meta-analysis was not performed as the included studies were not sufficiently homogeneous to provide a meaningful summary.

**Results:**

Six studies were identified as meeting the eligibility criteria. All studies were laboratory-based and used a repeated measures design. The quality of the studies varied, with Quality Index scores ranging from 7 to 10 out of 14. Five studies investigated foot orthoses and one studied in-shoe wedges. Foot orthoses were found to increase contact area in the midfoot, peak pressures under the hallux and were perceived to provide better arch support, compared to a control. With respect to physiological parameters, contrasting findings have been reported regarding the effect foot orthoses have on oxygen consumption. Further, foot orthoses have been shown to not provide effects on lower limb kinematics and perceived comfort. Both foot orthoses and in-shoe wedges have been shown to provide no effect on power.

**Conclusion:**

In general, there is limited high-quality research on the effects foot orthoses and in-shoe wedges provide during cycling. At present, there is some evidence that during cycling foot orthoses: increase contact area under the foot and increase plantar pressures under the hallux, but provide no gains in power. Based on available evidence, no definitive conclusions can be made about the effects foot orthoses have on lower limb kinematics and oxygen consumption, and the effect in-shoe wedges have on power during cycling. Future well-designed studies on this topic are warranted.

## Introduction

Cycling is typically known as a low weight bearing sport [1], yet a cyclist can apply forces of approximately half of their body weight to the pedal while cycling seated, and up to three times body weight while cycling standing [2]. Taking into account that a trained cyclist can average up to 5700 pedal revolutions in an hour [3], the interaction between the lower limb, foot and shoe-pedal interface requires consideration if one is to attempt to minimise injury and maximise performance during cycling.

Foot orthoses and in-shoe wedges have been advocated and used by cyclists to achieve a variety of goals [2,4]. Some of these goals include increasing comfort levels [2], injury prevention [5-7] and increasing power production [2,4,7]. The mechanism of action proposed to achieve these goals generally encompasses an improvement in the biomechanical alignment of the lower limb and foot, by seeking a more linear cycling motion [2,8,9]. This is believed to be especially beneficial in preventing overuse injuries of the knee [2,6,10-12] and improving power output in cyclists [2,4,7,13].

Despite the theoretical plausibility that foot orthoses and in-shoe wedges can provide a number of benefits during cycling, it is difficult to justify the use of such devices based on theories and anecdotal evidence alone. With the growing popularity of cycling [14,15], it is timely to summarise the effects these interventions provide cyclists. Therefore, the aim of this study was to perform a systematic review of the current literature by collecting, critically evaluating and summarising the effects foot orthoses and in-shoe wedges provide during cycling.

## Review

### Search strategy

This review aimed to summarise the literature on the effects foot orthoses and in-shoe wedges provide during cycling. A search of the following databases: Medline, CINAHL, Embase and SPORTDiscus were conducted to source relevant articles. In addition, Google Scholar was searched as an alternative source. The databases were searched using a standard search strategy and using a predetermined eligibility criteria (Table 1). The database search was conducted in the first week of February 2014. All publications that met the eligibility criteria had their reference lists hand searched for additional articles.

**Table 1:** Electronic database search strategy and eligibility criteria

Keywords used	(orthotic OR orthoses OR orthosis OR inner-sole OR insole OR wedges OR shims) AND (cycling or bicycling or cyclist or cycle) NOT (gait cycle)
Inclusion criteria	1) All empirical studies that evaluated the effects of foot orthoses, insoles, shoe inserts and in-shoe wedges on cycling.
	2) Outcome measures included kinematics and kinetics of the lower limb, physiological parameters and performance.
	3) Published in English language.
Exclusion criteria	1) Studies that examined pedal modifications only.
	2) Studies that examined orthoses or wedges external to the shoe.
	3) Studies that had subjects with significant or chronic disabilities or diseases that may affect the ability to cycle.
	4) Unpublished data.

### Methodological quality assessment

A quality assessment index of 14 items (maximum score of 14) was used to assess the methodological quality of each included study. The quality assessment was based on the Quality Index [16] and items relevant for laboratory-based studies were included. The original Quality Index scale, which consists of 26 items, has been shown to have high internal consistency (KR-20 = 0.89), test-retest (r = 0.88) and inter-rater (r = 0.75) reliability and high criterion validity (r ≥ 0.85) [16]. The two reviewers (BKY and DRB) independently scored the included studies using the index. Once all studies were scored, the reviewers met and discussed any discrepancies and a final score was obtained (Table 2).

**Table 2:** Modified Downs and Black Quality Index results for each study

	**1. Clear aim/ hypothesis**	**2. Outcome measures clearly described**	**3. Charateristics of patients included clearly described**	**4. Interventions of interest clearly described**	**6. Main findings clearly described**	**7. Measures of random variability provided**	**10. Actual probability values reported**	**11. Subjects asked to participate representative of population**	**12. Subjects prepared to participate representative of population**	**14. Blinding of subjects**	**15. Blinding of outcome assessor**	**16. Analyses performed were planned; no data dredging**	**18. Appropriate statistical tests used**	**20. Valid and reliable outcome measures**	**Total** ** (score out of 14)**
**Anderson & Sockler, 1990.**[19]	1	1	1	1	1	1	0	U	U	0	0	0	1	1	8
**Bousie et al., 2013.**[17]	1	1	1	1	1	1	1	U	U	0	0	1	1	1	10
**Dinsdale & Williams, 2010.**[4]	1	1	1	0	1	1	1	U	U	0	0	U	1	U	**7**
**Hice et al., 1985.**[18]	1	1	0	1	1	1	0	U	U	0	0	1	1	1	8
**Koch et al., 2013.**[7]	1	1	1	1	1	1	1	U	U	1	0	1	1	U	10
**O’Neill et al., 2011.**[20]	1	1	1	1	0	0	0	1	U	0	0	0	1	1	**7**

0: No, 1: Yes, U: Unable to be determined (received a score of 0).

### Statistical analysis

Data were synthesised descriptively. Meta-analysis was not considered as the included studies were not sufficiently homogeneous in terms of participants, interventions and outcomes to provide a meaningful summary.

## Results

**Figure 1:**
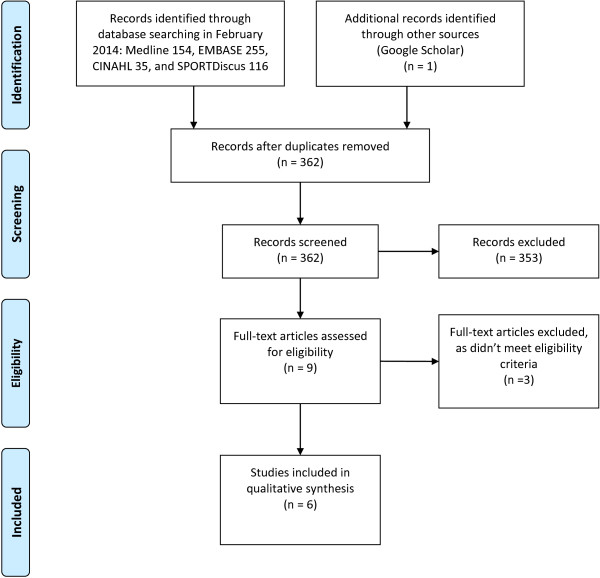
PRISMA flow diagram.

The search identified 362 potential titles and abstracts. Following screening, nine full-text articles were assessed for eligibility of which three were excluded. The remaining six studies were deemed suitable for inclusion [4,7,17-20] (Figure 1). Five of the studies investigated the use of foot orthoses [7,17-20] and one study investigated the use of in-shoe wedges [4]. All studies were laboratory-based and used a repeated measures design.

### Quality assessment of included studies

The quality of the six studies varied, with Quality Index scores ranging from 7 to 10 out of a possible 14 (Table 2). Three of the six studies did not report actual probability values [18-20], only one study attempted to blind participants [7] and no study blinded the assessors [4,7,17-20]. Two of the studies were judged as performing analysis that was unplanned [19,20], while it was unclear if this was the case with one other study [4].

All studies were considered as having used appropriate statistical tests to assess the main outcomes data; this was despite three studies with relatively small sample sizes using parametric tests [18-20]. However, as the distribution of data was not described [18-20], it was assumed to be distributed normally which is in adherence with the Quality Index [16]. Accordingly, the use of parametric tests was considered appropriate for these studies [18-20].

### The effects of foot orthoses during cycling

As previously stated, five studies investigated the effects foot orthoses provide during cycling [7,17-20]. Among the studies there were large variations in orthotic design and construction as three of the studies used custom-made foot orthoses [18-20] and two studies used pre-fabricated foot orthoses [7,17]. In addition, the studies used a variety of outcome measures as one analysed plantar pressures [17] and one studied hip and lower limb kinematics [20]. The remaining three studies investigated the effects foot orthoses have on cycling performance, with two studies investigating physiological parameters [18,19] and one focusing on power production [7].

Regarding cycling performance, the two studies that investigated the effects of custom-made foot orthoses on physiological parameters primarily focussed on oxygen consumption and found different results [18,19]. Hice and colleagues [18] reported that there was a statistically significant decrease in oxygen consumption (p < 0.05) when using orthoses compared to no orthoses. In contrast, the study by Anderson and Sockler [19] reported that orthoses provided no significant differences in oxygen consumption. The only study to investigate the effects of foot orthoses, which were pre-fabricated and cycling specific (Solestar GmbH, Berlin, Germany), on power production during cycling reported no significant difference on mean power (p = 0.68) and peak power production (p = 0.75) when compared with a non-contoured insole [7].

Compared to a control, the use of pre-fabricated foot orthoses have been shown to affect plantar pressures as significant increases in contact area in the medial (p = 0.0001; MD 5.7, 95% CI 3.0 to 8.4) and lateral (p = 0.009; MD 4.6, 95% CI 1.4 to 7.8) midfoot have been demonstrated [17]. Pre-fabricated orthoses also significantly increased plantar peak mean pressure under the hallux (p = 0.003; MD 21.4, 95% CI 9.1 to 33.6) [17]. In addition, the pre-fabricated orthoses was perceived to better support the arch (p < 0.001; MD 3.2, 95% CI 1.8 to 4.6) and heel region (p = 0.013; MD 1.3, 95% CI 0.3 to 2.3) compared to the control but no difference was reported for perceived comfort between conditions [17].

With respect to hip and lower limb kinematics, custom-made foot orthoses did not provide any statistically significant differences compared to no orthoses [20].

A summary of the findings from these five studies are presented in Table 3.

**Table 3:** Summary of the studies that have investigated the effects foot orthoses provide during cycling

**Author**	**Participants**	**Shoe, cleat and pedal characteristics**	**Intervention**	**Measures collected**	**Study design**	**Outcomes**
Anderson & Sockler, 1990. [19]	Ten healthy adult subjects (six males, four females).	Three males and three females wore stiff-soled cycling shoes with cleats. Four remaining subjects wore flexible-soled running shoes without cleats.	Participants were tested with custom-made foot orthoses (CFO) or without any orthoses. The CFOs were made from Rohadur®. Orthoses were molded using a non-weightbearing, netural position casting technique and included a rearfoot 4° inverted post and an intrinsic forefoot post with 4° motion.	Oxygen consumption, expired ventilatory volume, and heart rate.	Randomised, repeated measures, non-controlled study.	There were no significant differences in oxygen consumption, expired ventilatory volume, or heart rate between both conditions (p > 0.05).
	Mean age: 29.1 years (±2.1)					
	Mean height: 176.0 cm (±3.1)					
	Mean mass: 65.5 kg (±3.2)					
Bousie et al., 2013. [17]	Twelve competitive or recreational cyclists (eight males or four females).	Each participant wore their personal cycling specfic cleated road cycling shoes with a rigid sole, and used their personal pedals.	Participants used commercially available contoured orthoses and a flat non-contoured insert (Vasyli International Australia). Both orthoses and flat inserts were made of ethylene vinyl acetate (EVA), with the same hardness.	Plantar contact area, peak pressure, perceived comfort, and support of foot plantar surface.	Randomised, repeated measures, control study.	Compared to flat non-contoured inserts, the use of contoured orthoses led to a statistically significant increase in the contact area of the medial midfoot (p = 0.001; MD 5.7, 95% CI 3.0 to 8.4; SMD = 1.3) and lateral midfoot (p = 0.009; MD 4.6, 95% CI 1.4 to 7.8; SMD = 0.9). Contoured orthoses also produced a statistically significant increase in plantar pressures under the hallux (p = 0.003; MD 21.4, 95% CI 9.1 to 33.6; SMD = 1.1). Compared to the flat insert, the contoured orthoses was perceived to better support the arch (p < 0.001; MD 3.2, 95% CI 1.8 to 4.6; SMD = 1.5) and heel region (p = 0.013; MD 1.3, 95% CI 0.3 to 2.3; SMD = 0.9) but no difference was reported for perceived comfort.
	Mean age: 35.1 years (±10.6)					
	Mean height: 174.7 cm (±8.7)					
	Mean mass: 70.0 kg (±9.8)					
	Weekly riding distance: 285.4 km (±82.9)					
Hice et al., 1985. [18]	Five healthy adult cyclists (three males, two females) who cycle at least 3 hrs weekly.	All participants wore flexible soled shoes and used flat pedals.	Participants were tested with custom-made foot orthoses or without any orthoses. The CFO was made from rigid thermoplastic and were ¾ length. A neutral suspension casting technique was used to make the orthoses. Forefoot posting was applied to each CFO to achieve forefoot-rearfoot alignment.	Oxygen consumption and heart rate.	Non-randomised, repeated measures, non-controlled study.	A statistically significant decrease in oxygen consumption was found during the orthoses intervention when compared to no orthoses (p < 0.05). A decrease in heart rate was also observed when the subjects wore the orthoses compared to not wearing them, although only measurements at rest were statistically significant (p < 0.05).
Koch et al., 2013. [7]	Eighteen competitive male cyclists and triathletes.	There was no report of shoe, cleat and pedal characteristics that each participant used.	Participants were tested with cycling specific, commercially available, carbon-fibre cycling orthoses (Solestar, GmbH, Berlin) or non-contoured inserts.	Mean power production, peak power production.	Randomised, repeated measures, single blinded, controlled study.	There were no significant differences mean power production (p = 0.76) and peak power production (p = 0.53) between both conditions.
	Mean age: 26.3 years (±5.6)					
	Mean height: 181.9 cm (±4.7)					
	Mean mass: 76.7 kg (±4.4)					
	Foot length: 28.2 cm (±0.8)					
O’Neill et al., 2011. [20]	Twelve competitive cyclists (nine males and three females)	There was no report of shoe, cleat and pedal characteristics that each participant used.	Participants were tested with their own cycling-specific custom-made foot orthoses or without any orthoses. A variety of materials were used for each participant’s CFO, such as carbon fiber, polyvinyl alcohol (PVA) and plastic material. 10 of these orthoses were full length, while the remaining 2 were ¾ length. There were also a variety of modifications added to each orthoses, such as rearfoot and forefoot wedges, 1st metatarsophalangeal (MTPJ) cut out, metatarsal domes.	Maximum hip adduction, maximum knee abduction angle, total range of motion of tibial rotation, and coronal plane knee movement during the power phase of pedal stroke.	Non-randomised, repeated measures, non-controlled study.	No systemic effects from the CFOs were seen. Statistically significant subject specific effects, such as reduced tibial internal rotation motion, increased knee-to-bike distance and reduced knee abduction angle, from the CFO were reported (p < 0.05). All subjects had significant left to right leg differences during the power phase of pedalling.
	Males					
	Mean age: 40.0 years (±14.8)					
	Mean height: 179.4 cm (±7.6)					
	Mean mass: 82.7 kg (±8.0)					
	Cycling experience: 14.0 years (±9.7)					
	Females					
	Mean age: 29.0 years (±4.0)					
	Mean height: 169.7 cm (±7.3)					
	Mean mass: 63.6 kg (±7.5)					
	Cycling experience: 8.3 years (±3.1)					

Note: All available statistical data (i.e. mean differences, confidence intervals, p values) from the studies have been provided. All available information on shoe, pedal, cleat, and orthoses used by participants have also been provided.

*Abbreviations*: *MD* mean difference, *SMD* standardised mean difference (greater than 1.2 defined as large differences, 0.6 to 1.2 defined as moderate differences, and less than 0.6 defined as small differences).

### The effects of in-shoe wedges during cycling

As previously stated, one study investigated the effect that in-shoe wedges (forefoot varus) have on power production while cycling [4]. There was no significant difference in mean power (p = 0.10) or peak power production (p = 0.21) with and without forefoot varus wedges [4].

A summary of findings from this study are presented in Table 4.

**Table 4:** Summary of the study that investigated the effects in-shoe wedges provide during cycling

**Author**	**Participants**	**Shoe, cleat and pedal characteristics**	**Intervention**	**Measures collected**	**Study design**	**Outcomes**
Dinsdale & Williams, 2010. [4]	Six untrained males with a forefoot varus.	There was no report of shoe, cleat and pedal characteristics that each participant used.	Participants were tested with and without a forefoot varus wedge from commercial company, Specialized Bicycle Components. The size of the varus wedge (ranged between 1–4 degree) was customised to the size of each individual’s forefoot varus.	Mean power production, maximum power production, and anaerobic fatigue index.	Non-randomised, repeated measures, non-controlled study.	No significant difference in mean power production, maximum power production and anaerobic fatigue index (p = 0.10, p = 0.21, p = 0.24 respectively) between the two conditions.
	Mean age: 24.0 years (±5.0)					
	Mean height: 178.0 cm (±5.0)					
	Mean mass: 79.7 kg (±8.1)					
	Body fat: 10.3% (±3.2)					
	Forefoot varus: 6.1° (±1.7)					

Note: All available statistical data (i.e. mean differences, confidence intervals, p values) from the studies have been provided. All available information on shoe, pedal, cleat, and wedges used by participants have also been provided.

## Discussion

This systematic review evaluated the evidence surrounding the effects foot orthoses and in-shoe wedges provide during cycling. A number of complexities were encountered when reviewing the literature. These included variability of interventions and the materials used [7,17,20], differences in control interventions [7,17-19], differences in participant profiles [4,7,19,20] and diversity of footwear and shoe-pedal interfaces [17-19]. Furthermore, the outcome measures of the studies varied as some analysed kinetics [17], while others analysed kinematics [20], physiological parameters [18,19] or power production [4,7]. The methodological quality of the studies were generally low to moderate, especially in the domains of internal and external validity (Table 2). This is likely to influence the results of the studies and, as such, it is difficult to reach definitive conclusions based on the findings of some studies included in this review.

It is well documented that foot orthoses are regularly used and advocated in the management of overuse knee injuries [2,5,6,11,12], often based on the theory they provide a more linear cycling motion [8,9]. However, this is to be debated as there is no evidence supporting these effects. Only one study of low methodological quality investigated the effects of foot orthoses on lower limb kinematics and found no significant differences with and without foot orthoses [20]. Of interest, the authors reported significant subject-specific biomechanical effects (p < 0.05) produced by foot orthoses and therefore recommended an individualised approach in cycling orthotic prescription. Although this finding should be considered with caution due to the low quality of the study (Table 2) [20], it is consistent with the preferred motion pathway theory that foot orthoses elicit subject-specific responses [21]. Clearly, more high quality research is needed in this area to warrant the use of foot orthoses in altering lower limb kinematics as part of injury management in cyclists.

Of interest, the two studies that analysed physiological parameters, specifically oxygen consumption, with and without foot orthoses had opposing results [18,19]. The findings of both studies should be considered with some caution as they have a number of methodological limitations such as a lack of blinding and poor external validity (Table 2). In addition, the majority of subjects across the two studies used flexible running shoes and conventional flat pedals during data collection [18,19]. As modern cycling shoes are generally stiff soled and connect to the pedal via a cleat, it is likely that the findings from the aforementioned studies are not applicable to current competitive and recreational cyclists [22,23]. It is likely these differences would have a great influence on the overall results of the studies as it has been shown that these different shoe-pedal interfaces can significantly affect muscular activity [22] and sprint power output [24,25]. The stiffness of cycling shoes also provide significant differences in forefoot plantar pressures [23] and it is proposed that an adequate level of stiffness is required to efficiently transfer energy from the shoe to the pedal while cycling [2,26]. It is also noteworthy that although the subjects in both studies were regular cyclists, it is unclear as to their cycling ability [18,19]. This is an important consideration as competitive cyclists would most likely be the population interested in receiving physiological and performance gains from foot orthoses.

The effect forefoot varus wedges [4] and cycling specific carbon-fibre foot orthoses [7] have on power production during cycling has been investigated by two studies. The study by Dinsdale and Williams [4] suffered from several methodological limitations such as lack of randomisation, blinding of subjects and assessors and poor external validity (Table 2). The participants were untrained males and hence, like the studies analysing physiological parameters, the findings could not be generalised to trained or competitive cyclists. Of interest, the authors reported a significant Pearson’s correlation coefficient (p = 0.003; r = 0.0957) indicating that the use of forefoot varus wedges potentially provides greater mean power output for riders with greater degrees of a forefoot varus alignment. However, this finding needs to be interpreted in consideration of the small sample, the low methodological quality of the study, and the uncertainty of whether the correlation analysis was pre-planned and not a form of data dredging [4].

The study by Koch and colleagues [7] also investigated the effects of foot orthoses on power production and, consistent with the study by Dinsdale and Williams [4], found no significant difference between carbon foot orthoses and a sham device. This study [7] was of relatively high methodological quality (Table 2) and it had the distinguishing feature of being the only study included in this review that attempted to blind the subjects with a sham device. Importantly, and unlike the study by Dinsdale and Williams [4], the subjects used were trained competitive cyclists. However, the study [7] did not allow for a familiarisation period to the selected exercise protocol (Windgate Anaerobic Test) [27] and subjects are prone to fatigue while performing repeated bouts of the Windgate Anaerobic Test thus affecting the reproducibility and reliability of the results [28,29]. Crucially, the ergometer (Cyclus 2) used in the study [7] has not been shown to be a valid or reliable tool for measurement of power production [30,31]. It is possible that these factors may confound the results of the study.

The study by Bousie and colleagues [17] found foot orthoses were able to provide increased conformity to the plantar surface of the foot by increasing contact area under the midfoot. It would be intuitive to hypothesise that the increased conformity would result in greater comfort levels, yet no difference was found between the orthoses and the control [17]. It is possible that any potential benefit in comfort gained from the increased conformity was negated by the increase in pressure under the hallux [17]. These findings also need to be viewed in consideration that the plantar pressure and comfort data were collected in the same testing session as when the orthoses were initially issued [17]. In addition, the participants were instructed to cycled at a comfortable exertion level for a relatively short period of time [17]. Therefore, it is unclear if the effects provided by the orthoses on plantar pressures and comfort would be different following a longer acclimatisation period to the orthoses and if participants cycled at a higher intensity over a greater duration. The latter point is particularly important with respect to comfort as foot pain and paraesthesia has been reported to typically occur after an extended period of cycling [2,12,32]. It is interesting to note that despite the vastly different biomechanics in cycling compared to walking, the increased midfoot contact area with the use of foot orthoses during cycling [17] is consistent with the effect foot orthoses provide during walking [21,33,34].

There are unpublished data that investigated the effect of foot orthoses on lower limb kinematics [35,36], muscle activity [36] and power production [13] that would be relevant to this systematic review. However, as the studies and data have not undergone the process of peer review, it was not included in this review. In addition, a study by Baur and colleagues [37] was also found but was excluded from this review as only the abstract was published in English (Table 1 and Figure 1). Based on the abstract alone [37], carbon-fibre foot orthoses provided a statistically significant decrease in midfoot plantar peak pressure (p < 0.001) compared to a control condition, which is in contrast to the findings of Bousie and colleagues who reported no difference between orthoses and flat inserts [17]. However, as the full article by Baur and colleagues [37] was published in German, it is difficult to ascertain if the dissimilar findings are due to differences in the design of both studies as well as the plantar pressure variables investigated. Based on the abstract available by Baur and colleagues [37], the two studies [17,37] used different controls and different materials for the interventions. Of interest, both studies found an increase in peak pressure at the hallux when foot orthoses were compared to the control.

Finally, as high-quality research investigating the effects of foot orthoses and in-shoe wedges during cycling is generally lacking, future well-designed studies on this topic are justified. Future studies should attempt to incorporate attributes of high-quality evidence, many of which are checklist items on the Quality Index which was used in this study [16]. Taking into account the quality assessment of current studies included in this review, future studies should attempt to maintain high internal validity, such as blinding participants, and ensure high external validity is maintained. This latter point is particularly important as the study participants and the equipment used, such as footwear, pedals and bicycles, should be representative of the population being studied and those who the findings are likely to be applied to. In summary, it is essential that future studies are of relatively high methodological quality and are designed to be relevant to cyclists if they are to provide valuable information regarding the size of the effects foot orthoses and in-shoe wedges provide during cycling.

## Conclusions

There is limited research on the effects foot orthoses and in-shoe wedges provide during cycling. Present studies are generally of low to moderate methodological quality, vary in study design, and use different types of interventions and controls. Only three [7,17,20] of the six studies [4,7,17-20] included in this review used competitive cyclists and hence the findings of the other studies have low external validity in populations of competitive and elite cyclists. Despite the short-comings of some of the available literature, there is some evidence that foot orthoses increase contact area under the foot, increase plantar pressures under the hallux and provide no benefits in power production during cycling. Based on current evidence, no definitive conclusions can be made about the effects foot orthoses have on lower limb kinematics and oxygen consumption, and no definitive conclusions can be made regarding the effects in-shoe wedges may or may not provide during cycling. Future well-designed studies measuring the effects of foot orthoses and in-shoe wedges during cycling are warranted.

## Competing interests

The authors declare that they have no competing interests.

## Authors’ contributions

BKY and DRB were fully involved in the preparation and completion of the study procedures. BKY and DRB were responsible for the preparation and review of the manuscript prior to submission for publication. Both authors read and approved the final manuscript.
